# Clinical and prognostic significance of high-mobility group box-1 in human gliomas

**DOI:** 10.3892/etm.2014.2089

**Published:** 2014-11-25

**Authors:** XIN-JUN WANG, SHAO-LONG ZHOU, XU-DONG FU, YAN-YAN ZHANG, BO LIANG, JI-XIN SHOU, JIAN-YE WANG, JIAN MA

**Affiliations:** 1 Department of Neurosurgery, The Fifth Affiliated Hospital of Zhengzhou University, Zhengzhou, Henan 450052, P.R. China; 2 Department of Pathophysiology, College of Basic Medical Sciences of Zhengzhou University, Zhengzhou, Henan 450052, P.R. China

**Keywords:** high-mobility group box-1, gliomas, expression, clinical, prognosis

## Abstract

The objective of this study was to explore the expression and the clinical and prognostic significance of high-mobility group box-1 (HMGB1) in human gliomas. The expression of HMGB1 in 15 samples of normal brain tissue and 65 samples of different-grade glioma tissue was assayed using immunohistochemistry and western blot analysis. The associations between the differences in expression and pathology grades were analyzed statistically. Uni- and multivariate analyses were performed to investigate the prognostic value of HMGB1 expression and its expression levels. The positive rates of HMGB1 expression in normal brain and glioma tissue were 20.0% (3/15) and 76.9% (50/65), respectively. The expression of HMGB1 in glioma tissue was higher than that in normal tissue (P<0.05). The positive rates of HMGB1 expression in low-grade gliomas (LGGs, grades I and II) and high-grade gliomas (HGGs, grades III and IV) were 63.0% (17/27) and 86.8% (33/38), respectively, and the positive rates in HGG were higher than those in LGG (P=0.024). Western blot analysis showed that HMGB1 was also expressed in normal brain tissue. The expression levels in HGG were significantly higher than those in LGG (P<0.001). HMGB1-positive patients had significantly shorter overall survival times compared with HMGB1-negative patients (P=0.026). Increasing levels of HMGB1 expression significantly correlated with reduced survival times when all patients with glioma were considered (P=0.045). In conclusion, HMGB1 positivity and protein expression levels are of significant clinical and prognostic value in human gliomas. Detecting HMGB1 in human gliomas may be useful for assessing the degree of malignancy, and HMGB1 would appear to be a promising target in the clinical management of patients with glioma.

## Introduction

Gliomas are the most common type of central nervous system tumors, and the majority of the histological findings of the gliomas are malignant (1). Gliomas have obscure boundaries with the surrounding tissue and as a result the rate of radical resection is lower and the recurrence rate is higher than other intracranial tumors (2). In recent years with the use of microsurgical techniques and with the improvement of surgical skills, as well as radiation and chemotherapy, the overall survival of patients suffering from gliomas has been improved (3).

High-mobility group box-1 (HMGB1) is a non-histone DNA-binding protein that is widely present in tissues, including the heart, liver, lung, lymph, spleen, kidney and brain. In the liver and brain HMGB1 is primarily found in the cytoplasm; however, in other tissues it is mostly distributed in the nucleus (4). The functions of HMGB1 include DNA binding, stabilizing the nucleosome and regulating transcription (5). A number of studies have found that higher expression levels of HMGB1 are closely associated with tumor proliferation, invasion, migration and angiogenesis, as well as anti-apoptotic effects, and HMGB1 can attenuate the role of the body in monitoring tumor invasion and metastasis (6,7). Previous studies have found an increased expression of HMGB1 in human glioma tissues (8); however, the associations between expression levels, pathology grades and the prognostic significance are rarely reported.

At present studies of the molecular biology of tumors are key issues in order to explore the mechanisms of neoplasm occurrence and progress. In turn, the development of molecular biology can guide us in exploring new methods of glioma therapy, such as targeted therapy, which is a new and effective treatment method. HMGB1 has been found in most of human tumors and it is closely related with tumor development. HMGB1 may also play an important role in human gliomas, however related studies are rare.

In the present study, the expression of HMGB1 was examined in 15 samples of normal brain tissue and 65 samples of different-grade glioma tissue by immunohistochemistry and western blot analysis, and the associations between the expression level and pathology grades were analyzed statistically to investigate the clinical significance. To the best of our knowledge, this is the first study to investigate the prognostic significance of HMGB1 expression in human gliomas.

## Materials and methods

### Patients and samples

Tumor tissues were obtained at the first surgery in 65 previously untreated patients with glioma. The patient population comprised 39 males and 26 females, and the median age was 43.9±2.4 years (range, 12–78 years). All specimens were pathologically confirmed referring to the 2007 World Health Organization classification of tumors of the nervous system and grading criteria (9). Twenty-seven cases of low-grade glioma (LGG) were identified, including eight cases of pilocytic astrocytoma, six cases of diffuse astrocytoma, eight cases of oligodendroglioma and five cases of ependymoma. In addition, 38 cases of high-grade glioma (HGG) were identified, including 16 cases of anaplastic astrocytoma, two cases of anaplastic ependymoma, three cases of malignant oligodendroglioma, 15 cases of glioblastoma and two cases of medulloblastoma. Fifteen samples of normal brain tissue were obtained from brain injury decompression surgery as a control. Consent was received from the families of the patients to collect and preserve the specimens using cryopreservation at −80°C. The present study was approved by the Life Science Ethics committee of Zhengzhou University (Zhengzhou, China).

### Immunohistochemical detection

Immunohistochemistry was performed to detect the expression of HMGB1. The specimens were embedded, cut into serial 3-μm sections and placed on a slide after pretreatment that was undertaken by Beijing Bioss. In brief, pretreatment involved immersion of the slide in cleaning fluid of potassium dichromate sulfuric acid for 24 h and then rinsing under running water. After it was rinsed again at least three times with distilled water 95% ethanol was added and the slide was left to dry and immerse in poly-L-lysine (0.01%) for about 30 sec. Finally, the slide was drained and placed in the oven at 45°C for 1 h. Anti-HMGB1 monoclonal antibodies (1:20; Beijing Biosynthesis Biotechnology Co., Ltd., Beijing, China) were added to the sections for incubation overnight at 4°C, following by washing with phosphate-buffered saline (PBS) and incubation with biotin-labeled secondary antibody (Sigma-Aldrich, St. Louis, MO, USA) at room temperature for 20 min. Subsequent to further washing with PBS, horseradish peroxidase-labeled streptavidin working solution (Beijing Biosynthesis Biotechnology Co., Ltd.,) was added and the sections were incubated at room temperature for 15 min. 3,3′-diaminobenzidine chromogenic reagent was added for coloration, followed by rinsing, hematoxylin staining, conventional ethanol dehydration, xylene clearing, mounting with a neutral gum and observation under a microscope. Samples in which PBS displaced the primary antibody staining were classified as negative, while samples in which the cytoplasm was colored yellow or brown were classified as positive. An area with a strong immune response was selected in each slice, and five non-repetitive, high-power fields of view were observed (magnification, ×400). All controls provided satisfactory results. The immunohistochemical analysis was performed by one of the authors, who was blinded to the clinical data. The HMGB1-positive cells were counted, and the positive rate was calculated using the following formula: Positive rate = (number of positive cells/number of total cells) ×100%. The samples were defined as follows: Negative, 0–5%; weakly positive, 5–25%; positive, 26–50%; strongly positive, >50%.

### Western blot analysis

Frozen tissue samples (250 mg) were cut into sections, and then homogenized on ice with pre-chilled protein lysate (1 ml) for 30 min to fully cleave the HMGB1 protein. Homogenized tissue fluid was placed into 1.5-ml Eppendorf^®^ tubes (Eppendorf, Hamburg, Germany) and boiled for 5 min, and then centrifuged for 1 min at 8392 × g. Following centrifugation, the supernatant was removed into 200-μl Eppendorf tubes on ice and stored in a refrigerator at −70°C. The bicinchoninic acid kit (Shanghai GenePharma Co., Ltd., Shanghai, China) was used in the quantitative testing of HMGB1 protein levels. Samples were transferred to a polyvinylidene difluoride membrane following separation by SDS-PAGE and blocked with 5% skimmed milk overnight at 4°C. Subsequent to adding 1:400 rabbit anti-human HMGBl antibody (Beijing Biosynthesis Biotechnology Co., Ltd.), the membranes were again incubated overnight at 4°C. The secondary antibody was then added and incubated at 37°C for 1 h. A gel image analysis system (Media Cybernetics, Rockville, MD, USA) was used to determine the absorbance value of each band, which represented the expression of HMGB1 protein.

### Statistical analysis

All statistical analyses were performed using SPSS 17.0 software (SPSS, Inc., Chicago, IL, USA), and P<0.05 was considered to indicate a statistically significant difference. Measurement data are expressed as the mean ± standard deviation. Differences between two symmetrical portions of the same group of patients at different times were analyzed using the paired Student’s t-test. The correlation between HMGB1 expression and clinical pathological features was evaluated for statistical significance by χ^2^ and Fisher’s exact tests. Survival curves were calculated using the Kaplan-Meier method. The log-rank test was used to analyze the survival time for any significant differences.

## Results

### Analysis of the expression of HMGB1 in normal brain tissue and gliomas

**Figure 1:**
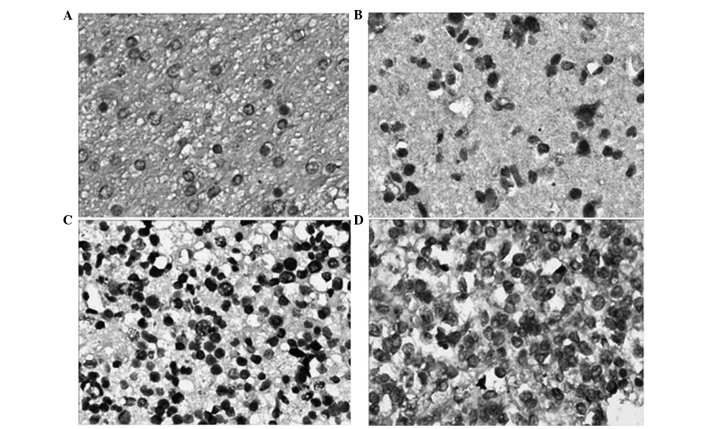
Expression of high-mobility group box-1 in the different grades of gliomas (streptavidin-peroxidase staining; magnification, ×400). (A) Normal brain tissue; (B) astrocytoma (grade II); (C) oligodendrocyte tumors (grade III); (D) glioblastoma (grade IV).

Immunohistochemical staining showed that the colored areas due to HMGB1 expression were mainly located in the cytoplasm near the nucleus. The majority of the staining was pale yellow, while darkly stained areas were brown or tan (Fig. 1). HMGB1 showed lower expression in the 15 normal brain tissue samples, and the positive expression rate was 20.0% (3/15). However, in the 65 glioma samples the HMGB1 positive expression rate was 76.9% (50/65), which was higher than that in the normal brain tissue (P<0.05) (Table I). The difference in HMGB1 expression between the different gender and age groups was not statistically significant (P>0.05). The positive expression rate of HMGB1 in the LGG and HGG groups was 63.0% (17/27) and 86.8% (33/38), respectively, and the difference between the two groups was statistically significant (χ^2^=5.070, P=0.024) (Table II).

### Analysis of the expression levels of HMGB1 by western blotting

**Figure 2:**
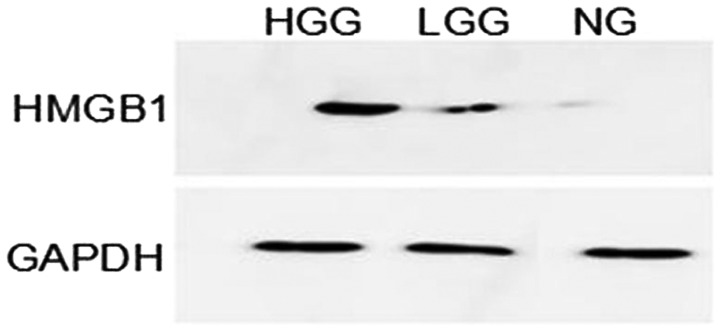
Western blot analysis of normal brain and glioma tissues. GAPDH was used as the internal reference. NG, normal group; LGG, low-grade glioma; HGG, high-grade glioma; HMGB1, high-mobility group box-1.

Western blot analysis showed that HMGB1 had lower levels of expression in normal brain tissue than in glioma tissue. The HMGB1 band optical density of each specimen was compared with that of GAPDH and the ratio represented the relative levels of HMGB1 protein expression. Analysis showed that the data were consistent with a normal distribution; therefore, analysis of variance was used for the three sets of data (normal, LGG and HGG). The difference was found to be statistically significant (P<0.05). The least significant difference t-test showed that the expression of HMGB1 in the HGG group was significantly higher than that in the LGG group (P<0.001) (Table III). A representative western blot analysis of HMGB1 expression levels from identical cases is shown in Fig. 2.

### Prognostic value of HMGB1 positivity

**Figure 3:**
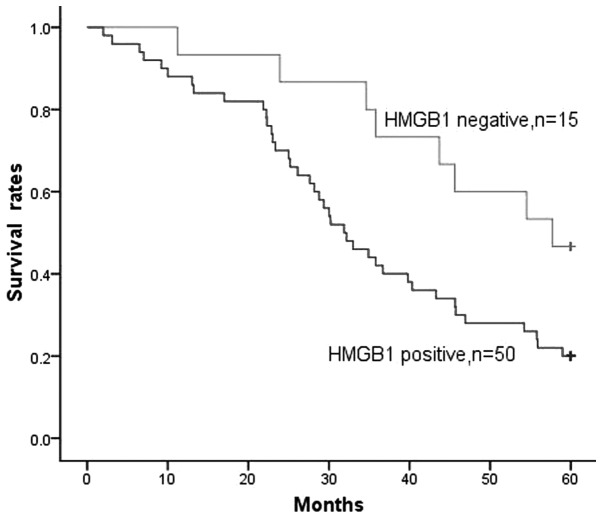
Overall survival rates by HMGB1 expression for all gliomas. Patients with HMGB1-positive glioma were shown to have significantly shortened survival times compared with patients with HMGB1-negative glioma (P=0.026). Of the 65 patients with glioma, immunohistochemical detection revealed that 50 were HMGB1-positive (bottom curve) and 15 were HMGB1-negative (top curve). HMGB1, high-mobility group box-1.

HMGB1 expression was detected in the majority of patients, with 50/65 (76.9%) of gliomas being HMGB1-positive. When all patients with glioma were considered together, the survival rate of patients with HMGB1-negative tumors was significantly higher than that of patients with HMGB1-positive tumors (P=0.026, Fig. 3). The survival time of patients with HMGB1-negative tumors was 48.4±4.0 months, compared with 35.2±2.6 months for patients with HMGB1-positive tumors.

### Prognostic value of HMGB1 expression levels

**Figure 4:**
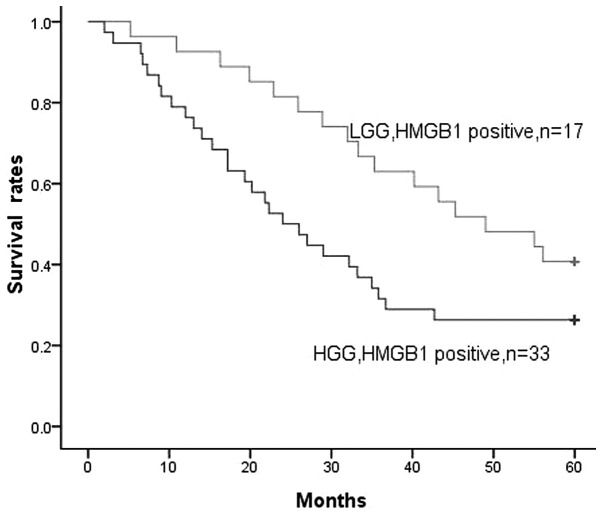
Significant association between HMGB1 expression levels and overall survival rates of patients. A significantly negative correlation was identified between the intensity of HMGB1 expression and survival rate. There appeared to be a stronger correlation with reduced survival times for patients with HGG than for patients with LGG (P=0.045). LGG, low-grade glioma; HGG, high-grade glioma; HMGB1, high-mobility group box-1.

As stated earlier, a positive correlation was identified between HMGB1 expression levels and the pathological grades of the gliomas. In the LGG and HGG groups, positive HMGB1 expression was found in 17 and 33 cases, respectively. When all gliomas were considered, increasing levels of HMGB1 expression were clearly associated with decreased survival time. There appeared to be a stronger correlation with reduced survival time for patients with HGG than for patients with LGG (P=0.045, Fig. 4). HMGB1 expression levels had a significant association with decreased survival time for patients with glioma. This suggests that HMGB1 expression at the highest levels is associated with a more extreme malignant phenotype of glioma and may also be associated with increased treatment resistance.

### Multivariate analysis of prognostic factors

In a model that included the presence or absence of HMGB1 expression, pathological grade (LGG versus HGG), gender and age, pathological grade (P=0.037) and HMGB1 expression (P=0.021) were significantly associated with reduced survival times, whereas age and gender were not. The data are presented in Table IV.

## Discussion

Glioma is the most common type of intracranial tumor and has the highest incidence and mortality. The adult incidence rate is ~6/100,000 and the five-year survival rate for glioma is 20–30% (10,11). Glioma exhibits the biological features of infiltrative growth and unclear boundaries; therefore, total resection is difficult. Additionally, the tumor recurs easily. Although comprehensive treatments, including radiotherapy and chemotherapy, are available, the curative effect requires improvement (12). HMGB1 may be an important factor involved in the processes of glioma occurrence and development, and may seriously affect the prognosis of patients with glioma.

HMGB1, which is a highly conserved nuclear protein, functions as a chromatin-binding factor. As such, HMGB1 bends DNA and facilitates access to transcription and protein assembly on specific DNA targets. The functions of HMGB1 also include stabilizing the nucleosome and regulating transcription (5). HMGB1 is expressed in the nucleus and cytoplasm and plays an important role in the chemoresistance of glioma, in addition to acting as a broad-spectrum tumor biomarker (13). HMGB1 is passively released from necrotic cells and actively secreted by inflammatory cells, and functions as an extracellular signaling molecule during processes such as inflammation, cell differentiation, tumor cell proliferation, cell migration and tumor metastasis (14,15). HMGB1 has been revealed to be constitutively activated in a wide variety of human tumor tissues and cell lines, including colorectal, breast, lung, prostate, cervical, stomach and liver cancer, as well as leukemia (16). It has been suggested that the overexpression of HMGB1 may promote certain genes to form a tumor phenotype, rendering the cells immune to apoptosis and resulting in tumorigenesis (17). The mechanism by which HMGB1 is involved in tumorigenesis is unclear. However, it is believed to mainly include the activation of the Janus kinase (JAK)/signal transducer and activator of transcription (STAT) signaling pathway, which occurs through HMGB1 binding with high affinity to several receptors, including the receptor for advanced glycation end products (RAGE), Toll-like receptor (TLR)-2, TLR-4 and TLR-9. These interactions trigger the activation of key signaling pathways involved in the regulation of cell differentiation, growth, motility and apoptosis. A number of studies have revealed that HMGB1 can overactivate STAT by activating the JAK/STAT pathway. Activated STAT, particularly STAT3, inhibits tumor cell apoptosis, accelerates the cell cycle and thus leads to tumorigenesis (18–20). Subsequent to HMGB1 being released into the extracellular environment, it unites with its high affinity receptor RAGE and upregulates RAGE expression (21). HMGB1-RAGE interactions activate mitogen-activated protein kinase and protein kinase B signaling pathways, resulting in extracellular matrix degradation, tumor invasion and metastasis, leading to tumor development (22). A previous study (23) found that HMGB1 that is released into the extracellular environment may cause surrounding tumor cells to undergo constant proliferation and induce the regeneration of small blood vessels, thus promoting tumor growth. HMGB1 is also closely associated with tumor drug resistance. A previous study found that HMGB1 induces autophagy, causing the cells to become resistant to chemotherapy drugs (24).

As a broad-spectrum tumor marker, the abnormal expression of HMGB1 contributes to the occurrence and development of numerous types of tumor (25). In the present study it was shown that HMGB1 has different levels of expression in normal brain and glioma tissues. Normal brain tissues were weakly positive for HMGB1, which could be due to HMBG1 acting as a DNA-binding protein involved in normal physiological processes of the body (26). In glioma tissues the expression of HMGB1 was higher, and the degree of expression in different pathological grade gliomas was significantly different. HMGB1 is believed to be a pivotal factor in the association between necrosis and malignancy in glioma due to its role as an autocrine factor, which can promote the growth and migration of tumor cells (27). HMGB1 may cause disordered gene expression, resulting in glial cells obtaining a tumor phenotype and resistance to apoptosis, and ultimately leading to tumorigenesis (28). A recent study indicated that necrotic cells can release HMGB1 into the extracellular environment (29), and necrosis is a characteristic feature of malignant gliomas. With the consistent expression of HMGB1, the glioma grows and progresses continually, leading to necrosis of certain lesions. The necrotic tumor cells secrete HMGB1 to cause a cycle of further tumor progression. Finally, the tumor infiltrates the surrounding brain tissue and presents a stronger resistance, which makes it difficult to attain whole resection and causes poor prognosis (30).

In the present study, immunohistochemistry and western blot analysis were used to analyze the expression rate and levels of HMGB1 in glioma tissues, and the prognostic significance of HMGB1 expression in human gliomas was examined for the first time. It was revealed that the expression of HMGB1 was associated with pathological grade and poor prognosis. HMGB1 expression patterns may lend additional insight into the molecular pathogenesis of these tumors. Furthermore, HMGB1 may be an important prognostic marker. Therefore, treatments targeting HMGB1 are expected to become a novel therapeutic approach towards the treatment of patients with glioma.

## Figures and Tables

**Table I:** Expression rates of HMGB1 in normal brain and glioma tissues.

		HMGB1 expression			
				
Group	n	Positive, n (%)	Negative, n (%)	χ^2^	P-value
NG	15	3 (20.00)	12 (80.00)	-	-
LGG	27	17 (62.96)	10 (37.04)	7.136	0.008^a^
HGG	38	33 (86.84)	5 (13.16)	-	<0.001^a^,^b^

Compared with NG,

a P<0. 05;

b Fisher’s exact test.

NG, normal group; LGG, low-grade glioma; HGG, high-grade glioma; HMGB1, high-mobility group box-1.

**Table II:** Association between HMGB1 and the clinicopathological factors of glioma.

		HMGB1 expression			
				
Variable	n	Positive, n (%)	Negative, n (%)	χ^2^	P-value
Gender					
Male	39	33 (84.62)	6 (15.38)	3.250	0.071
Female	26	17 (65.38)	9 (34.62)		
Age in years					
>45	43	35 (81.40)	8 (18.60)	1.431	0.232
≤45	22	15 (68.18)	7 (31.82)		
Pathological grade					
LGG	27	17 (62.96)	10 (37.04)	5.070	0.024
HGG	38	33 (86.84)	5 (13.16)		

LGG, low-grade glioma; HGG, high-grade glioma; HMGB1, high-mobility group box-1.

**Table III:** Western blot analysis detecting HMGB1 expression levels in normal brain and glioma tissues.

Group	n	OD_HMGB1/GAPDH_
NG	15	0.3631±0.1429
LGG	27	0.9115±0.1562^a^
HGG	38	1.7019±0.1581^a^,^b^

Compared with NG,

a P<0. 001; Compared with LGG,

b P<0. 001.

NG, normal group; LGG, low-grade glioma; HGG, high-grade glioma; HMGB1, high-mobility group box-1; OD, optical density.

**Table IV:** Multivariate analysis of prognostic factors.

Variable	B	SE	χ^2^	P-value	OR	95% CI
Gender	0.124	0.562	0.049	0.835	0.394	0.135–1.291
Age	−0.521	0.669	0.918	0.376	0.608	0.197–1.917
HMGB1 expression	−2.961	0.864	11.558	0.021	0.061	0.019–0.294
Pathological grades	−1.734	0.814	4.083	0.037	0.188	0.035–0.957

HMGB1, high-mobility group box-1; SE, standard error; OR, odds ratio; CI, confidence interval.
